# Analyzing mHealth Engagement: Joint Models for Intensively Collected User Engagement Data

**DOI:** 10.2196/mhealth.6474

**Published:** 2017-01-12

**Authors:** Emily A Scherer, Dror Ben-Zeev, Zhigang Li, John M Kane

**Affiliations:** ^1^ Department of Biomedical Data Science Dartmouth Geisel School of Medicine Hanover, NH United States; ^2^ Psychiatry, Neurology, and Neuroscience Hofstra Northwell School of Medicine Hepstead, NY United States; ^3^ Department of Psychiatry Zucker Hillside Hospital Glen Oaks, NY United States

**Keywords:** joint models, engagement, informative missingness

## Abstract

**Background:**

Evaluating engagement with an intervention is a key component of understanding its efficacy. With an increasing interest in developing behavioral interventions in the mobile health (mHealth) space, appropriate methods for evaluating engagement in this context are necessary. Data collected to evaluate mHealth interventions are often collected much more frequently than those for clinic-based interventions. Additionally, missing data on engagement is closely linked to level of engagement resulting in the potential for informative missingness. Thus, models that can accommodate intensively collected data and can account for informative missingness are required for unbiased inference when analyzing engagement with an mHealth intervention.

**Objective:**

The objectives of this paper are to discuss the utility of the joint modeling approach in the analysis of longitudinal engagement data in mHealth research and to illustrate the application of this approach using data from an mHealth intervention designed to support illness management among people with schizophrenia.

**Methods:**

Engagement data from an evaluation of an mHealth intervention designed to support illness management among people with schizophrenia is analyzed. A joint model is applied to the longitudinal engagement outcome and time-to-dropout to allow unbiased inference on the engagement outcome. Results are compared to a naïve model that does not account for the relationship between dropout and engagement.

**Results:**

The joint model shows a strong relationship between engagement and reduced risk of dropout. Using the mHealth app 1 day more per week was associated with a 23% decreased risk of dropout (*P*<.001). The decline in engagement over time was steeper when the joint model was used in comparison with the naïve model.

**Conclusions:**

Naïve longitudinal models that do not account for informative missingness in mHealth data may produce biased results. Joint models provide a way to model intensively collected engagement outcomes while simultaneously accounting for the relationship between engagement and missing data in mHealth intervention research.

## Introduction

The success of a behavioral intervention depends upon participants’ active engagement in treatment. Engagement with treatment is a multifaceted state with behavioral, affective, and cognitive components that contribute to maximizing positive treatment outcomes [1]. Treatment engagement is therefore a key component of any evaluation of treatment efficacy. With an increasing interest in developing behavioral interventions in the mobile health (mHealth) space [2], appropriate methods for evaluating engagement in this context are necessary. Indeed, evaluating engagement in mHealth has been identified as critical for improving the impact of technology-based mental health interventions [3,4].

Unlike clinic-based care, mHealth data are often collected much more intensively [5], allowing more detailed patterns to emerge in the outcomes of interest [6]. With mHealth interventions, engagement evaluations usually focus on the behavioral component and examine various measures of mHealth intervention usage [3,7]. Outcome data may be available daily if quantified as app usage, short message service (SMS) messaging, passive sensing data, response to prompts, or use of an online portal, for example. More so than in a single time point, we must consider the nature of missing data in intensively collected engagement outcomes. Furthermore, compared with other clinical outcomes, engagement is particularly likely to have missing data related to the outcome value itself. For example, if a participant is disengaged in treatment and thus unlikely to attend a therapy session, there is an increased likelihood that the participant does not return for a follow-up visit as well. In the mHealth context, the problem is compounded in that mode of follow-up data collection and intervention delivery is often the same. That is, the collection of an intensively collected engagement outcome like app usage is directly tied to engagement itself. The availability of engagement data is likely strongly related to level of engagement with the intervention. Therefore, missingness in engagement outcomes should be considered to be nonrandom and nonignorable [8,9].

Longitudinal models such as mixed effects models and latent growth curve models are robust to random missingness but not to nonrandom missingness like that likely present in longitudinal engagement data [8,10]. That is, failure to take into account the mechanism of missingness results in biased inference about the outcome [11,12]. Time-to-dropout and longitudinal engagement are linked processes, and examining either separately is likely to miss key information. Analyzing intensively collected engagement therefore requires longitudinal methodology that takes into account nonrandom missing data. The model must also accommodate flexible patterns of engagement over time which can be captured when so many data points are available. Using a joint model enables simultaneous modeling of the longitudinal outcome and the dropout mechanism to accommodate data missing not at random. Models that jointly evaluate the time-to-event and longitudinal processes have previously been shown to reduce bias in estimation of the effects in the longitudinal and time-to-even processes [13-16]. They have been successfully applied in nonintensive, longitudinal studies (as in Henderson et al [14], for example). These models, however, have not previously been applied in intensively collected data in the mHealth context where they are particularly relevant.

Recent work has highlighted the need to understand engagement with mHealth interventions with the goal of designing effective interventions that meet users’ needs [1,7]. Levels of engagement with an mHealth intervention may change over time and have important implications for understanding the success of an intervention. Understanding how engagement changes over time, factors associated with changes in level of engagement, and how engagement is related to changes in behavior targeted by the mHealth intervention could inform intervention tailoring and improvement. Therefore, accurate estimation of behavioral engagement over time is essential.

The objectives of this paper are to discuss the utility of the joint modeling approach in the analysis of longitudinal engagement data in mHealth research and illustrate the application of this approach using data from an mHealth intervention designed to support illness management among people with schizophrenia. We use data from a large implementation study (ClinicalTrials.gov NCT02364544) which involved the use of a smartphone intervention (FOCUS) designed to support illness management among people with schizophrenia. The study data, described in detail in a separate article [17], consist of weekly engagement outcomes. We first introduce both longitudinal and time-to-event submodels that make up the joint model. We then illustrate the need for joint modeling by examining the difference in observed engagement outcome by amount of available data. After performing a naïve analysis of the data that does not take into account nonrandom missingness, we analyze and interpret the engagement data via joint modeling and contrast the results of the 2 approaches.

## Methods

### FOCUS Intervention Analysis

The data for this evaluation are from a multisite implementation project that recruited participants at 10 community mental health centers and outpatient clinics. Eligible participants were individuals between the ages of 18 and 60 years with psychotic disorders who had recently been discharged from a psychiatric hospitalization. Participants were offered a technology-assisted relapse prevention program that could last up to 6 months. Variation in program duration was due to both participant-related (eg, discontinued phone use and/or study follow-ups) and project-related (eg, funding ended) factors. As part of the program, participants were provided with a smartphone with the FOCUS illness self-management program installed. FOCUS consists of both prompted (3 times per day) and self-initiated use where each use starts with a brief self-assessment and is followed by educational/intervention content. Program discontinuation was identified when participants notified study staff of a desire to end participation and/or returned the study phone. In addition, when participants enrolled in the last 5 months of the study, they participated for less than a full 6 months. Finally, when participants stopped generating phone data, stopped attending in-person services, and study staff were unable to contact them after repeated attempts, the study team made the determination of discontinuation.

The evaluation of engagement with the FOCUS intervention assessed the decline of engagement over time for this long-term mHealth intervention as well as factors that may be associated with differing rates of decline. Curvilinear declines were seen in each engagement outcome: Days of mHealth Use, Days Responding to Prompts, Days of On-Demand Use, and Daily On-Demand Use. In addition, several demographic and psychiatric variables were found associated with longitudinal engagement. Models of time to dropout included gender, age, and race as potential predictors [17]. In the current demonstration of joint modeling, we focus on the research question of change in engagement over time using Days of mHealth Use per week as the engagement outcome.

### Joint Model Set-Up

Joint models are comprised of 2 submodels: the longitudinal model of a continuous outcome and a time-to-event model. Using notation from Rizpoulous [18], the observed longitudinal outcome for individual *i*, *y_ij_* is observed multiple times, *j*=*1*,...,*n_i_*. The longitudinal submodel is a linear mixed effects model

*y_i_*(*t*)=*x′_i_*(*t*)***β***+*z′_i_*(*t*)***b**_i_*+*ε_i_*(*t*),

where ***β*** is a vector of fixed effect regression coefficients associated with the predictors *x_i_*(*t*) and the vector ***b**_i_* is a set of individual-level random effects associated with predictors *z_i_*(*t*). We assume a normal distribution for both ***b**_i_* and *ε_i_*(*t*), (***b**_i_*~*N* (0,*D*), *ε_i_*(*t*)~*N*(0,*σ*^2^)), and also that these 2 random variables are independent of each other. In this application, the outcome, *y_i_*(*t*), is engagement measured as weekly mHealth intervention usage. The research question is whether engagement changes over the course of the study, so time from randomization, a quadratic effect of time, and a fixed intercept term are included in *x_i_*(*t*). Other flexible models of time are possible, but for simplicity, we focus on this parametric model which appears to fit the observed trajectory well. For other research questions, other predictors may be included in *x_i_*(*t*). Due to the focus on changes over time, we have included only time variables in the longitudinal model in this application, but it is straightforward to include additional variables in this model including the baseline predictors used in the time-to-event model. In *z_i_*(*t*), we include a random intercept and slope term. The model of engagement is therefore:


*y_i_*(*t*)=*β*_0_+*β*_1_*t*+*β*_2_*t*^2^+*b*_0*i*_+*b*_1*i*_*t*+*ε_i_*(*t*). [Equation 1]

We rewrite the above equation in a different format in order to introduce the term *m_i_*(*t*), which represents the true value of the longitudinal outcome for individual *i* at time *t*, measured without error:

*y_i_*(*t*)=*m_i_*(*t*)+*ε_i_*(*t*).

Time-to-event models are referred to as survival models, as they are often applied to survival data that is only fully observed in some participants (those who die while in the study). In the behavioral sciences, time-to-event models can be applied to model times to any event where the event may not be observed in all individuals (eg, time to relapse or time to recovery). When the study ends prior to an individual’s relapse to smoking, that participant’s time to relapse is only partially observed. That is, it is known that he or she remained abstinent for the duration of the study, but the time of relapse is unknown. These partially observed times are said to be censored. In the context of engagement, the partially observed time-to-event data is the time to dropout. Time-to-dropout data is fully observed among those participants who drop out prior to the end of study. Time to dropout is censored when the study follow-up period ends.

The time-to-event submodel is given as a proportional hazard model [19]:


*h_i_*(*t*|*w_i_*,*m_i_*(*t*))=*h*_0_(*t*)exp{***γ**′w_i_*+*αm_i_*(*t*)}

Importantly, the true value of the longitudinal trajectory, *m_i_*(*t*), is a predictor in this model representing the assumption that the longitudinal trajectory influences the risk of dropout. Other baseline covariates in the model are represented by *w_i_*. In the current application, we include available baseline predictors that may influence the time to dropout: age, gender, and race (black, Hispanic, and other with white as the reference group):


*h_i_*(*t*|*w_i_*,*m_i_*(*t*))=*h*_0_(*t*)exp{*γ*_1_*age*+*γ*_2_*male*+*γ*_3_*race_Black_* +*γ*_4_*race_Hisp_*+*γ*_5_*race_Oth_*+*αm_i_*(*t*)} [Equation 2]

The semiparametric proportional hazard model does not require an assumption about the distribution of the time to event, and the parameter estimates associated with predictors in the model are conveniently interpreted as hazard ratios. For example, being male is associated with a risk of dropout that is exp{*γ*_2_} times the risk of dropout in females.

Estimation of the parameters in each model is performed by maximizing the log likelihood of the joint distribution of the longitudinal and time-to-event outcomes [18]. This joint model is known as a shared parameter model since the parameters that define the individual-level trajectory (random and fixed effects) influence both the longitudinal trajectory and the time-to-event model. Thus, the random effects account for both the association between the longitudinal and time-to-event outcomes and the nonindependence of repeated observations within individual [18].

### Joint Modeling of Engagement

Nonignorable missingness, or missingness not at random (MNAR), occurs when the probability of missingness depends on unobserved longitudinal responses [8,11]. That is, it occurs if certain values of a variable are more likely to be missing than other values. In the case of engagement, it is very likely that lower levels of engagement are less likely to be observed because a participant who becomes less engaged over time is much more likely to drop out of the study. Longitudinal engagement data is therefore particularly subject to informative missingness. In the current study, engagement, defined as the number of days in a week that the participant used the mHealth intervention, is collected each week for up to 6 months. Participants provided data for differing amounts of time ranging from less than 1 month to 6 months or more. A participant who provided less than 6 months of data is considered to have dropped out for the purpose of the time-to-event analysis. This happened for various reasons. In some cases, the reason is administrative and is likely not informative (ie, value of the unobserved data should not be viewed as related to the data that would have been observed); for example, mobile data collection stopped because the implementation effort came to an end. On the other hand, there are several participants who stopped providing mobile data before the study ended. In the latter case, we should assume that the value of the engagement outcome that would have been observed (ie, if the participant provided data) is lower.

If we knew that all participants who dropped out did so due to disengagement (eg, stopped participating or using the phone due to lack of interest in the intervention), it might be reasonable to impute a 0 value for engagement for all weeks postdropout. This would be considered a worst-case scenario as it is possible that had these participants not dropped out they would have had some engagement even if it were low. However, there are also cases where dropout is unrelated to engagement, including administrative dropout or moving out of the area, lost phone, etc. For these 2 reasons, we should not assume that all missing data represents the worst case scenario of complete disengagement. The joint model allows for a relationship between level of engagement and likelihood of dropout but does not make assumptions that all missing data represents a complete lack of engagement. In this way, the joint model flexibly handles dropout that may or may not be related to engagement.

To implement the joint model, we used the JM package in R [18] (R Project). The model estimated is described in equations 1 and 2 above. Naïve models for longitudinal outcome and time-to-dropout were fit via linear mixed effects models and Cox proportional hazard models, respectively, using the lme function in the nlme package [20] and the survfit function in the survival package [21] in R.

## Results

Data from 342 participants who used the FOCUS intervention for at least 1 week were included in these analyses. The mean age of this sample was 35 (SD 11) years; 62.3% were male, 50.0% were white, 25.2% were African American, 10.8% were Hispanic, and the remaining 14.0% reported being Asian, American Indian, Native Hawaiian, or more than one race.

**Figure 1:**
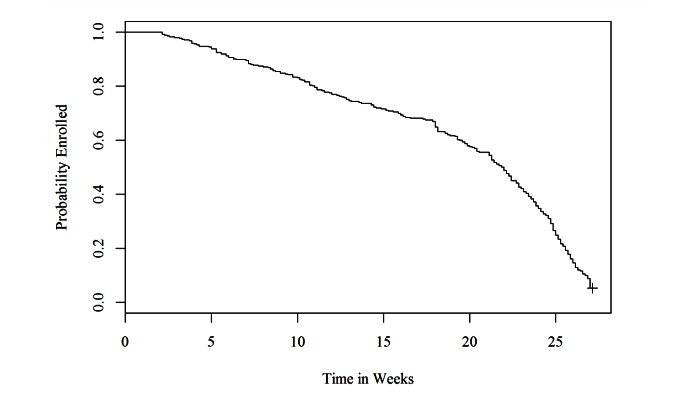
Kaplan-Meier estimate of probability of duration of mobile data availability over the course of the study.

Kaplan-Meier estimates of the time-to-dropout are presented in Figure 1. Median time-to-dropout in this study was 22 weeks, but dropout occurred throughout the course of the study. After a participant dropped out, engagement data were no longer available.

**Figure 2:**
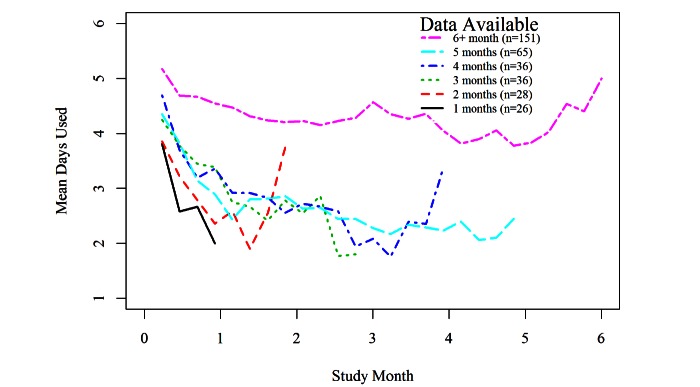
Mean engagement with mHealth intervention (intervention use) over the course of the study for groups of participants categorized by duration of mobile data provided.

To illustrate the relationship between level of engagement and amount of data provided, we grouped participants by duration of mobile data provided. At each time point the available data within each group are used to compute a mean engagement. Figure 2 illustrates that participants who provided the most data for the longest duration had the highest level of engagement. Likewise, participants who discontinued using the intervention after only 1 month had a very low level of engagement during the time they were actually providing data. One of the benefits of mixed effects models is that data are not required at all time points for all participants. This is possible because the model estimates an individual’s trend over time based on the data from that individual augmented by the trend of the full sample of participants [22]. However, this is problematic in the context of nonignorable missing data. If during the later months, data are only available from those participants who provided data for several months and those participants tended to be more engaged throughout, estimates from a naïve model during the later months will rely on data provided by highly engaged participants and therefore overestimate the level of engagement at those times.

**Figure 3:**
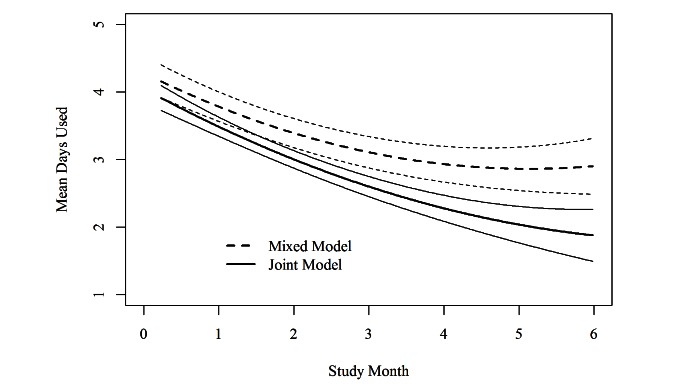
Model-based estimated mean engagement with mHealth intervention (intervention use) over the course of the study. Estimates (and 95% confidence intervals) from the joint model of engagement and time-to-drop-out and from the naïve mixed effects model not accounting for drop-out are displayed.

The longitudinal engagement outcome is Days of mHealth Use per week (range 0-7). Sometimes count variables can be considered to have a Poisson distribution, but unlike a Poisson random variable, the distribution of this variable was symmetric around the mean (not skewed) and somewhat kurtotic. There is evidence supporting the consideration of Likert scale variables with multiple categories as continuous variables [23], and mixed effects models have been shown robust to both non-Gaussian random effects distributions [24,25] and non-Gaussian residual errors [26]. We therefore examined the distribution of the longitudinal engagement variable and the residuals from the mixed effects model to assess the appropriateness of the longitudinal submodel for this engagement outcome. Both indicated that there was not a significant deviation from normality and the model-based estimates fit the raw data means well. Table 1 and Figure 3 show the results of a naïve mixed effects model of engagement not taking into account dropout alongside the results when the joint model is implemented. The longitudinal models are similar with significant linear and quadratic terms showing a significant decline in engagement over time (negative linear time term) that is steeper toward the beginning of the study and levels off as the study progresses (negative quadratic time term). Figure 3, however, shows that the mixed model estimates a higher level of engagement than the joint model and this difference is pronounced toward the end of the study. At baseline, estimated level of engagement in the 2 models differs only by about 0.2 days per week. By 6 months, however, the model-estimated engagement from the naïve model is 2.9 days per week of uses, whereas the model-estimated engagement from the joint model is 1.8 days per week, a difference of 1.1 days per week.

Examining the naïve time-to-dropout model versus the time-to-dropout submodel of the joint model that includes longitudinal engagement as a predictor, we see that no baseline covariates have a significant effect on time-to-dropout in either model, but it is clear in the joint model time-to-dropout submodel there is a strong association between engagement level and risk of dropout. Specifically, using the mHealth intervention 1 day more per week is associated with 0.77 (exp (−0.26)) times the risk of dropout at any time (*P*<.001). That is a 23% decreased risk of dropout associated with greater engagement.

**Table 1:** Model results from separate models of engagement with an mHealth intervention (defined as intervention use) and time to dropout and the joint model of engagement and time to dropout.

		Separate models		Joint model	
		Parameter estimate	*P* value	Parameter estimate	*P* value
**Longitudinal engagement outcome**					
	Intercept (*β*_0_)	4.28 (0.13)	<.001	4.05 (0.10)	<.001
	Study week 1 (*β*_1_)	−0.13 (0.014)	<.001	−0.14 (0.014)	<.001
	Study week 2 (*β*_2_)	0.0029 (0.0005)	<.001	0.0021 (0.0006)	.001
**Time-to-dropout**					
	Age (*γ*_1_)	0.084 (0.14)	.58	−0.021 (0.13)	.87
	Male (*γ*_2_)	0.073 (0.12)	.54	−0.10 (0.10)	.32
	Black versus white (*γ*_3_)	0.058 (0.14)	.67	−0.097 (0.13)	.46
	Hispanic versus white (*γ*_4_)	0.12 (0.19)	.51	0.15 (0.18)	.41
	Other versus white (*γ*_5_)	0.27 (0.17)	.10	0.11 (0.17)	.49
	Longitudinal engagement association (*α*)			−0.26 (0.022)	<.001

## Discussion

Examining intensively collected engagement with the mHealth behavioral intervention made clear that level of engagement varied by amount of available mobile data. Naïve mixed effects models of engagement showed a slight decrease over the 6-month course of the study, but these results weight data from highly engaged participants toward the end of the study period leading to possibly biased results. Joint modeling of the linked processes of engagement and time to dropout allowed for an examination of engagement over time that more appropriately accounted for missing engagement data. These model results indicated a greater decline in engagement with the mobile intervention over time in the population. Furthermore, the time-to-event submodel of the joint model specifically quantifies the association between longitudinal engagement and dropout. The association is seen to be statistically significant, with those who are more engaged significantly less likely to drop out. And conversely, those who are less engaged are much more likely to drop out and therefore much more likely to yield missing engagement outcome data.

The present analysis represents just 1 example of implementing a joint model and comparing it to a naïve mixed model for engagement with an mHealth intervention. However, similar patterns between models would be expected assuming an association between increased likelihood of missingness and lower engagement. That is, the joint model results will likely estimate lower levels of engagement than a naïve mixed model. The magnitude of the difference between results from a mixed model and longitudinal submodel of a joint model depends on the association between engagement and missing data in the particular dataset being analyzed, the level of missing data, and the pattern of missingness over time. Therefore, a comparison of models from a different dataset may produce different results.

While missing data in the context of longitudinal studies is always a concern, often this missingness can be handled with the usual longitudinal modeling techniques such as mixed effects models. Importantly, with engagement data, the assumptions necessary for valid inference from typical models are likely not met since level of engagement may be related to likelihood of missing data. In this case, typical longitudinal models produce biased results. It is therefore especially important to account appropriately for missing data in analyses of engagement outcomes. With mHealth interventions, engagement is collected more intensively and often in the same mode as treatment is delivered so addressing missing engagement data is especially important. In the current investigation, we focus only on the behavioral component of engagement as this is frequently measured intensively via mobile devices and therefore most relevant for the modeling concepts presented.

Joint models are straightforward to implement with the JM package in R and offer flexibility in modeling the longitudinal trajectory over time. While in the current application we only used parametric models of time (quadratic), more flexible patterns of change over time can be accommodated by using spline basis terms in the longitudinal submodel of the joint model. Parametric assumptions on the time-to-event data are also not required.

There are other types of shared parameter models that model the longitudinal and/or time-to-event data differently with respect to specifying the individual-level trends in the longitudinal outcome, specifying the dependence of the time-to-event processes on these individual-level trends, varying the form of the time-to-event model, and approaching the estimation of model parameters [12]. The shared parameter model implemented in the current application is that proposed by Wulfsohn and Tsiatis [16]. Other methods for modeling longitudinal data with dropout, including random coefficient selection models and random coefficient pattern mixture models, are summarized in Little [27]. Pattern mixture models [22,28] estimate separate longitudinal trajectories by groups defined by dropout time and summarize the trajectory for the population by averaging the groups. When a limited number of dropout patterns are present to define the groups or when the goal is to examine trajectories separately by time of dropout, pattern mixture models may be most appropriate and also can be easily implemented. Related to pattern mixture models, the terminal decline model [29] is geared toward examining the longitudinal trajectory just prior to dropout or death. Selecting an appropriate model to accommodate nonignorable missingness is important and should be geared toward the research question. The shared parameter joint model implemented here is especially appropriate for intensively collected longitudinal data because the focus is on examining the longitudinal trajectory of the population over time, the pattern of engagement can be modeled flexibly, grouping individuals by dropout time is unnecessary, and no assumption is made about the distribution of the time to dropout.

Assessing engagement with mHealth behavioral interventions is crucial to evaluating their efficacy. Modeling intensively collected engagement should be done via models that appropriately account for the potential of nonignorable missing data. Using the shared parameter joint model implemented in the JM package in R is a straightforward way to flexibly model intensively collected engagement data like that from mHealth interventions and to examine the relationship between engagement and missing data.

## Abbreviations

MNAR: missingness not at random
SMS: short message service
